# Shape differences in the semitendinosus following tendon harvesting for anterior cruciate ligament reconstruction

**DOI:** 10.1002/jor.25337

**Published:** 2022-04-17

**Authors:** William du Moulin, Matthew Bourne, Laura E. Diamond, Jason Konrath, Christopher Vertullo, David Lloyd, David J. Saxby

**Affiliations:** ^1^ Griffith Centre of Biomedical and Rehabilitation Engineering (GCORE), Menzies Health Institute Queensland Griffith University Gold Coast Queensland Australia; ^2^ Principia Technology Crawley Western Australia Australia; ^3^ Knee Research Australia Gold Coast Queensland Australia

**Keywords:** muscle morphology, muscle segmentation, regional differences, shape similarity, tendon regeneration

## Abstract

Following hamstring autograft anterior cruciate ligament reconstruction (ACLR), muscle length, cross‐sectional area, and volume are reduced. However, these discrete measures of morphology do not account for complex three‐dimensional muscle shape. The primary aim of this study was to determine between‐limb semitendinosus (ST) shape and regional morphology differences in young adults following tendon harvest for ACLR and to compare these differences with those in healthy controls. In this cross‐sectional study, magnetic resonance imaging was performed on 18 individuals with unilateral ACLR and 18 healthy controls. Bilaterally, ST muscles were segmented, and shape differences assessed between limbs and compared between groups using Jaccard index (0–1) and Hausdorff distance (mm). Length (cm), peak cross‐sectional area (cm^2^), and volume (cm^3^) were measured for the entire muscle and proximal, middle, and distal regions, and compared between limbs and groups. Compared to healthy controls, the ACLR group had significantly (*p* < 0.001, Cohen's *d* = −2.33) lower bilateral ST shape similarity and shape deviation was significantly (*p* < 0.001, *d* = 2.12) greater. Shape deviation was greatest within the distal region of the ACLR (Hausdorff: 23.1 ± 8.68 mm). Compared to both the uninjured contralateral limb and healthy controls, deficits in peak cross‐sectional area and volume in ACLR group were largest in proximal (*p* < 0.001, *d* = −2.52 to −1.28) and middle (*p* < 0.001, *d* = −1.81 to −1.04) regions of the ST. Overall, shape analysis provides unique insight into regional adaptations in ST morphology post‐ACLR. Findings highlight morphological features in distal ST not identified by traditional discrete morphology measures. Clinical significance: Following ACLR, risk of a secondary knee or primary hamstring injury has been reported to be between 2‐to‐5 times greater compared to those without ACLR. Change in semitendinosus (ST) shape following ACLR may affect force transmission and distribution within the hamstrings and might contribute to persistent deficits in knee flexor and internal rotator strength.

## 1 INTRODUCTION

Following anterior cruciate ligament (ACL) rupture, reconstruction (ACLR) is typically recommended to restore knee stability.^1^ In Australia, ~90% of ACLR harvest semitendinosus (ST), alone or in combination with gracilis (GR), for tendon autografts.^2^, ^3^ Following ACLR, harvested ST and/or GR tendons partly regenerated in ~70% of patients.^4^ Although athletes return to sport after ACLR,^1^ long‐term deficits in knee flexor and internal rotator strength persist,^5^, ^6^ due in part to postoperative morbidity of harvested muscle. Muscle morbidity is traditionally assessed through discrete morphological measures (i.e., length, cross‐sectional area [CSA], volume). However, the full extent of morphological maladaptation post‐ACLR may not be well described by these discrete measures as they do not assess the complex three‐dimensional shape of muscle.

The ST is digastric, long, thin, and fusiform,^7^ and theoretically well suited to high shortening velocities and large excursions.^8^, ^9^ A knee flexor and hip extensor, ST is also an important antagonist of external knee valgus moments which is recruited to support the knee during tasks such as landing and change of direction commonly performed during field and court sports and associated with ACL rupture.^10^, ^11^, ^12^ Following tendon harvest for ACLR, ST experiences atrophy,^5^, ^6^ retraction,^13^ fatty infiltration,^14^ and impaired voluntary activation.^15^ Post‐ACLR strength deficits have been correlated with ST CSA and volume, indicating ST morphology affects its functional capacity in this clinical population.^5^, ^16^ Chronic deficits in ST CSA and volume have been observed as long as 7‐years after ACLR, despite partial donor tendon regeneration in most patients.^17^ Compared to intact ST, regenerated ST tendon tends to insert more proximal and medial on the tibia, within the popliteal fascia, and, in some cases, may not regenerate.^4^, ^18^ Altered ST morphology after ACLR may reduce torque generating capacity of the hamstrings via reductions in muscle force‐generating capacity, moment‐arm, or both. Quantifying the three‐dimensional shape of the ST may better describe structural and functional consequences following ACLR.

Shape modeling has been used to study human anatomy following cardiac,^19^ orthopedic,^20^ and complex maxillofacial surgeries,^21^ and to investigate musculoskeletal structures in the lower limb^22^ and foot.^23^ To date, no study has evaluated three‐dimensional muscle shape differences between limbs for those following hamstring ACLR. The aims of this study were to (1) determine bilateral ST shape similarity and regional morphology in those with ST‐GR ACLR and compare to healthy controls; and (2) in those with an ACLR, determine bilateral ST shape similarity and regional morphology in those with and without postoperative tendon regeneration. We hypothesized (1) bilateral ST shape similarity would be lower in those with an ACLR when compared to healthy controls, and regional morphology differences would be more pronounced in the distal region of ST. Additionally, (2) within the ACLR cohort those with ST tendon regeneration when compared to those with no ST regeneration will have greater bilateral ST shape similarity and regional muscle morphology.

## 2 METHODS

### 2.1 Participants

This level 3 cross‐sectional study involved a secondary analysis of previously published data from individuals with a history of ACLR^5^ and healthy controls.^22^, ^24^ As this was the first study to explore shape modeling of the ST following ACLR, it was not possible to base sample size estimates on previous reports. Instead, the secondary outcome of muscle volume was used. Previous studies have reported effect size of 1.52^6^ and 1.71^25^ when comparing surgically reconstructed to uninjured contralateral limbs and to healthy controls, respectively. Therefore, conservative sample size estimates were based on an anticipated effect size of 0.8 and a sample size of 12 was deemed sufficient to provide a statistical power of ≥0.8 when *p* < 0.05. All available data were used thereby increasing the total study population to 18 ACLR and 18 healthy participants.

For ACLR cohort, inclusion criteria were: (1) an isolated ACL rupture sustained without any other concomitant knee ligament injury, (2) an ACLR with the use of a quadrupled autologous ST‐GR tendon graft within the previous 2–4 years, (3) aged between 18 and 45 years, and (4) the ability to comply with the testing protocol. Exclusion criteria were (1) any contraindications to magnetic resonance imaging (MRI), (2) complex knee injuries with additional ligament surgery or meniscal injury, and (3) previous ACL or lower extremity surgery. All surgical reconstructions were performed by one senior orthopedic surgeon (CV) using the same protocol. For healthy control cohort, 18 volunteers recruited from Griffith University above 18 years of age and met the inclusion/exclusion criteria were included. These criteria included a verbal medical history questionnaire administered via telephone to determine if participants suffered from any medical conditions, such as musculoskeletal, neurological, or other health problems that may have affected their ability to participate. Ethics approval was obtained through Griffith University Research Ethics committee (reference: PES/36/10/HREC & GU Ref No: 2017/521), with all participants providing their written informed consent before any testing.

### 2.2 MRI

A 3‐Tesla MRI scanner (Philips Medical Systems, Australia) was used to acquire images of both lower limbs in all participants. Participants were instructed to lie supine in atop the scanner bed, and images were acquired from the level of the iliac crest to the ankle mortise. Axial T_1_‐weighted three‐dimensional fast field echo sequences were performed with a slice thickness of 2.4 mm with a 0.5 mm interslice gap. Voxel size was 1.2 × 1.3 × 1.2 mm and field‐of‐view was 300 × 452 mm.

### 2.3 MRI processing

**Figure 1:**
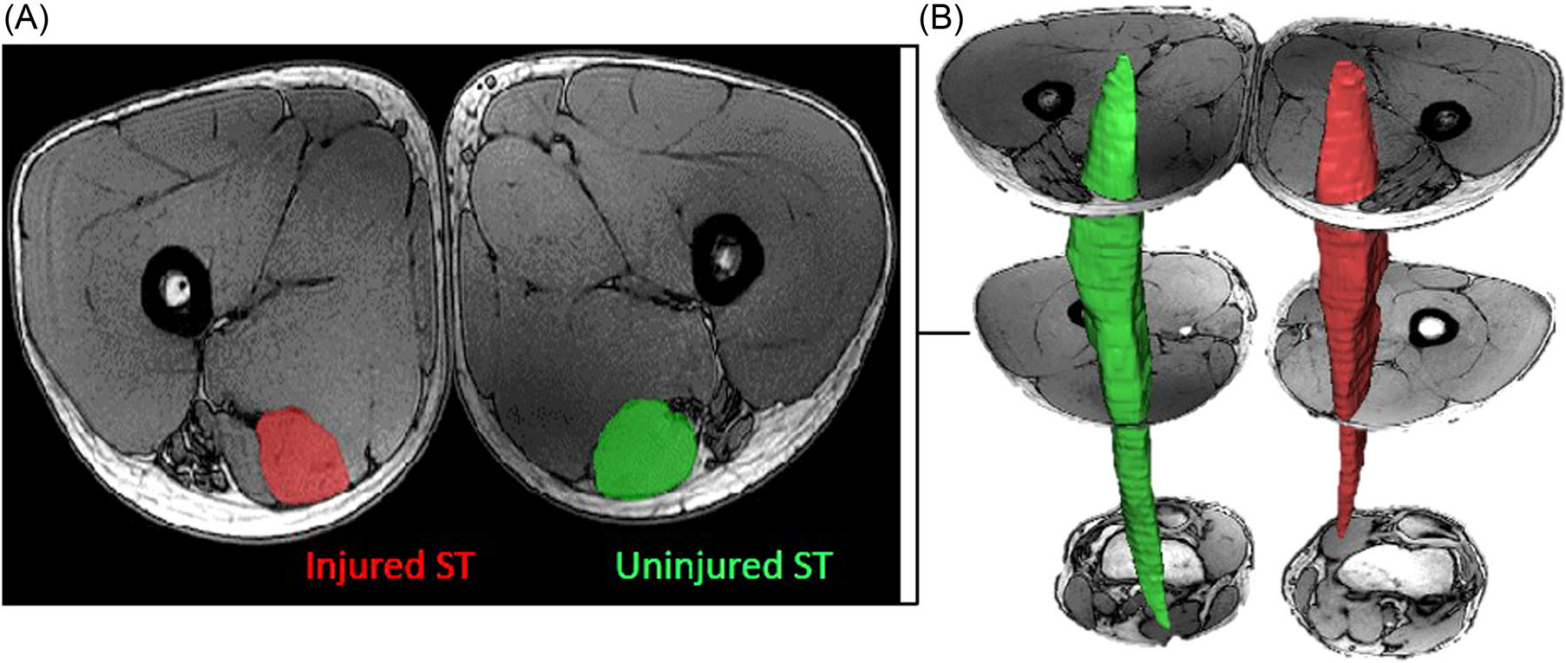
Muscle segmentation from a participant with unilateral anterior cruciate ligament reconstruction involving a semitendinosus (ST)‐gracilis (GR) autograft. (A) Cross‐sectional magnetic resonance image displaying the surgically harvested and uninjured contralateral ST. (B) The ST was segmented manually from magnetic resonance images to calculate muscle length, peak cross‐sectional area, and volume. In this image, 3 of 489 segmented slices are depicted. [Color figure can be viewed at wileyonlinelibrary.com]

All MRI data were processed and analyzed using the Materialise Interactive Medical Image Control System software (Mimics, Materialise, v21). Within each axial slice of the fast field echo images, the outer boundaries of muscles were visualized and traced as separate objects. These traced margins were then used to create a three‐dimensional mesh model of the ST muscle using the calculate part tool within Mimics (Figure 1). There was excellent^26^ inter/intra‐rater reliability for muscle segmentation volume (Intraclass correlation coefficient [ICC] = 0.997; 95% confidence interval [CI]: 0.86–0.99 and ICC = 0.991; 95% CI: 0.86–0.99, respectively). A wrapping factor was applied to each mesh model with a gap closing distance of 1.3 mm and smallest detail of 0.82 mm followed by a smoothing factor of 0.4. Distal tendon regeneration was defined as having occurred if the neo‐tendon was visible below the distal muscle‐tendon junction and traced to the level of the femoral epicondyle. Two subgroups from the ACLR cohort were established: (1) regenerated and (2) nonregenerated.

### 2.4 Volumetric shape modeling

To prepare the muscle meshes for volumetric shape similarity measurements between limbs, meshes were exported to 3‐Matics Research (Materialise, v13). In healthy controls, a right‐sided muscle was mirrored to the left. In the ACLR cohort, the injured muscle was mirrored to the uninjured side. To focus on shape difference rather than gross size differences between limbs in the ACLR cohort, the injured muscle was linearly scaled to approximate the length of the healthy contralateral muscle. The two muscle meshes were then rigidly aligned using N‐point registration within 3‐Matics Research using 3 points on the proximal musculotendinous junction (MTJ) and 3 points on the distal MTJ systematically selected by the user. A part comparison analysis was conducted within 3‐Matics Research to produce a heatmap illustrating areas of greatest difference between the two meshes. The aligned meshes were then exported to Python (Python Software Foundation, v2.7) to perform shape similarity assessment.

### 2.5 Shape similarity analysis

Shape similarity between injured and uninjured (ACLR) and left and right (healthy controls) ST used the following metrics: Jaccard index (0–1), root mean square error (RMSE; mm), and Hausdorff distance (mm). Jaccard index quantifies volumetric similarity as the ratio of intersection between two registered volumes (e.g., left limb vs mirrored right limb) relative to the union of those two volumes. A Jaccard index of 1 indicates perfectly overlapping volumes. The RMSE is a measure of average error between the two ST volumes. Hausdorff distance is the maximum distance between two similar points on the two aligned ST volumes. The spatial coordinates of the maximum Hausdorff distance were also extracted from the aligned ST volumes to identify the location on the muscle where greatest between‐limb difference was located.

### 2.6 Discrete regional morphology analysis

Using the Measure and Analyze toolboxes within the Mimics software, muscle length (cm) and volume (cm^3^) were calculated from the wrapped and smoothed muscle meshes. Fit Centreline tool was used to calculate the centroid of the muscle or tendon boundary for each axial slice. These centroids were then linked and linearly interpolated to create the intercentroid pathway, which represented the total length of the respective muscle or tendon. The CSA at any given point along the intercentroid pathway was determined by fitting a plane to the outer boundary of the muscle but orthogonal to the pathway. This process was performed across the length of the pathway for each muscle with the largest value being recorded as peak CSA (cm^2^). Muscle regions were defined by percentage of muscle length: proximal (100%–66%), middle (<66%–33%), and distal (<33%–0%). Morphological measures were normalized to limb length (m) for muscle length (cm^.^m^−^¹) and mass (BW) (kg) x limb length (m)^27^ for peak CSA (cm²^.^kg^−^¹^.^m^−^¹), and volume (cm^3.^kg^−^¹^.^m^−^¹), respectively.

### 2.7 Statistical analysis

All statistical analyzes were performed using SPSS v27 (IBM Corp.). Results are presented as mean ± standard deviation (SD) for parametric variables, and frequencies and proportions for categorical or binary variables. Bilateral shape similarity measures (Jaccard index, RMSE, and Hausdorff distance) were compared between ACLR and healthy control cohorts using independent *t*‐tests. A mixed‐design MANOVA was used to assess interaction between group (Control/ACLR) and limb (left/right or uninjured/injured) for total and regional (proximal, middle, distal) discrete morphology variables (length, peak CSA, and volume). When significant interactions were detected, post hoc pairwise comparisons were used. If no significant difference between healthy control limbs was found, all subsequent analysis used an average of the measure across two limbs. To evaluate postoperative tendon regeneration, regenerated and non‐regenerated subgroups were compared for bilateral ST shape similarity and all discrete morphology measures (total and regional) assessed using independent *t*‐tests. For all analyzes, significance was set as *p* < 0.05. For all significant findings, mean difference, 95% CI, and Cohen's *d* were calculated. Cohen's *d* was reported as a measure of effect size, with effect levels deemed small (*d* = 0.20–0.49), medium (*d* = 0.50–0.79), or large (*d* > 0.80).^28^

## 3 RESULTS

### 3.1 Participant demographics

Compared to controls, the ACLR cohort was significantly heavier (12.66 [95% CI: 5.27–20.05] kg; *p* = 0.001; *d* = 0.81) but did not differ in age or limb length (Table 1). Within the ACLR cohort, 39% (7/18) of harvested ST tendons regenerated (i.e., distal neo‐tendon visible below distal muscle‐tendon junction and traced to femoral epicondyle).

**Table 1:** Participant characteristics

	Controls	Anterior cruciate ligament reconstruction
	(*n* = 18)	(*n* = 18)
Age (years)	29.60 ± 4.99	28.84 ± 7.79
Males, *n* (%)	10 (52)	13 (68)
Mass (kg)	69 ± 15.4	82 ± 15.6*
Limb length (m)	0.81 ± 0.05	0.83 ± 0.07
Regenerated tendon, *n* (%)	N/A	7 (39)

*Note*: Data reported as mean ± standard deviation unless otherwise indicated.

* *p* < 0.05; compared with healthy control cohort.

### 3.2 Shape similarity

**Figure 2:**
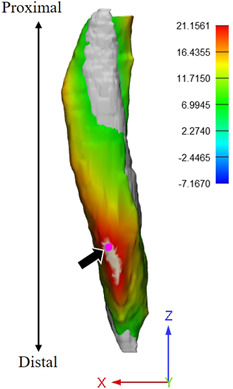
A semitendinosus (ST) muscle (colored) from a participant with anterior cruciate ligament reconstruction (ACLR) is scaled and then aligned to the homologous muscle in their uninjured contralateral limb (gray). Distance between muscles is represented by the color map which ranges from ‐10 mm (blue) to 25 mm (red). The pink point (highlighted with arrow) is the coordinate of the maximum Hausdorff distance (mm). [Color figure can be viewed at wileyonlinelibrary.com]

Compared to healthy controls, the ACLR cohort had significantly lower bilateral ST shape similarity (Table 2). The shape of ST from the ACLR leg substantially deviated (Hausdorff distance 23.1 ± 8.68 mm) from the shape of the healthy contralateral ST, particularly within the distal region (Figure 2). Compared to those in the ACLR cohort with non‐regenerated ST tendon, those with regenerated ST in the ACLR cohort had significantly more bilateral shape similarity as indicated by higher Jaccard (0.17 [95% CI: 0.08–0.25]; *p* = 0.001; *d* = 1.97), lower RMSE (−3.66 [95% CI: −5.36 to −1.97] mm; *p* < 0.001; *d* = −2.2), and lower Hausdorff distance (−14.14 [95% CI: −19.31 to −8.96] mm; *p* < 0.001; *d* = −2.74).

**Table 2:** Shape similarity outcome measures^a^

	Healthy (*n* = 18)	ACLR (*n* = 18)	Mean difference	95% CI		Cohen's *d*
				Lower bounds	Upper bounds	
Jaccard, 0‐1	0.70 ± 0.09	0.47 ± 0.12*	0.24	0.17	0.31	2.33
RMSE, mm	3.34 ± 1.03	7.57 ± 2.44*	−4.24	−5.51	−2.97	−2.26
H‐distance, mm	9.60 ± 2.47	23.1 ± 8.68*	−13.5	−17.83	−9.18	−2.12

^a^ Data reported as mean ± 1 standard deviation unless otherwise indicated; ACLR, anterior cruciate ligament; RMSE, root mean squared error; H‐distance, max Hausdorff distance (mm); 95% CI, 95% confidence interval of the mean difference.

* *p* < 0.001; ACLR cohort significantly different to healthy control cohort.

### 3.3 Discrete muscle morphology (complete muscle)

**Figure 3:**
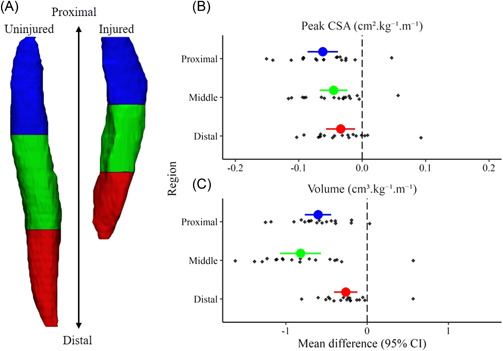
Mean difference of regional morphological measures in anterior cruciate ligament reconstruction (ACLR) participant (injured–uninjured). (A) Regional segments from a representative ACLR participant depicting semitendinosus (ST) muscle length in proximal (blue 100%–66%), middle (green < 66%–33%), and distal (red < 33%–0%) regions, (B) normalized peak cross‐sectional area (CSA), and (C) normalized muscle volume across each region. CI: Confidence interval of the mean difference. [Color figure can be viewed at wileyonlinelibrary.com]

A significant group‐by‐limb (*p* < 0.001) interaction was found for all morphology measures. Compared to uninjured contralateral ST, ACLR ST was significantly shorter (−12.45 [95% CI: −14.52 to −10.38] cm^.^m^−1^; *p* < 0.001; *d* = −2.99), smaller in peak CSA (−0.47 [95% CI: −0.70 to −0.02] cm²^.^kg^−^¹^.^m^−^¹; *p* < 0.001; *d* = −1.03), and of less volume (−1.68 [95% CI: −2.20 to −1.17] cm^3.^kg^−^¹^.^m^−^¹; *p* < 0.001; *d* = −1.63) (Figure 3, Table 3.). Compared to healthy controls, ACLR ST was significantly shorter (−10.21 [95% CI: −13.56 to −6.85] cm^.^m^−1^, *p* < 0.01, *d* = −2.06), smaller in peak CSA (−0.05 [95% CI: −0.08 to −0.02] cm²^.^kg^−^¹^.^m^−^¹, *p* < 0.001, *d* = −1.6), and of less volume (−1.49 [95% CI: −2.01 to −0.97] cm^3.^kg^−^¹^.^m^−^¹, *p* < 0.001, *d* = −1.96). There were no significant bilateral differences in ST length, peak CSA, and volume in healthy controls. No between‐group differences were found for ST length, peak CSA, and volume between the uninjured contralateral limb and healthy control limbs.

**Table 3:** Discrete muscle morphological outcomes^a^

	Average healthy	Uninjured ACLR limb	Injured ACLR limb
	(*n* = 18)	(*n* = 18)	(*n* = 18)
Length, cm.m^−1^	36.64 ± 3.01	38.89 ± 3.85	26.44 ± 6.32*, **
Peak CSA, cm². kg⁻¹.m⁻¹	0.18 ± 0.04	0.18 ± 0.04	0.13 ± 0.05*, **
Volume, cm^3^. kg⁻¹.m⁻¹	3.21 ± 0.58	3.40 ± 0.75	1.72 ± 0.90*, **
Regional Peak CSA, cm². kg⁻¹.m⁻¹			
Proximal region	0.16 ± 0.04	0.17 ± 0.04	0.10 ± 0.04*, **
Middle region	0.18 ± 0.04	0.18 ± 0.04	0.13 ± 0.04*, **
Distal region	0.14 ± 0.03	0.14 ± 0.05	0.1 ± 0.04*, **
Regional volume, cm^3^. kg⁻¹.m⁻¹			
Proximal region	1.00 ± 0.21	1.01 ± 0.33	0.45 ± 0.22*, **
Middle region	1.57 ± 0.31	1.66 ± 0.35	0.85 ± 0.48*, **
Distal region	0.64 ± 0.14	0.69 ± 0.23	0.42 ± 0.23*, **

^a^ Data reported as mean ± 1 standard deviation unless otherwise indicated; Average healthy, average of the left and right limb of the healthy control cohort was reported.

* *p* < 0.05 compared to healthy controls

** *p* < 0.05 compared to the uninjured contralateral limb.

### 3.4 Regional differences in muscle morphology

A significant group‐by‐limb (*p* < 0.001) interaction was observed for all regional muscle morphology measures.

#### 3.4.1 Proximal region

Compared to uninjured contralateral limb ST, proximal ACLR ST was significantly smaller in peak CSA (−0.06 [95% CI: −0.09 to −0.04] cm²^.^kg^−^¹^.^m^−^¹, *p* < 0.001, *d* = −1.28) and volume (−0.60 [95% CI: −0.77 to −0.44] cm^3.^kg^−^¹^.^m^−^¹, *p* < 0.001, *d* = −1.85) (Figure 3). Compared to healthy controls, ACLR ST was significantly smaller in peak CSA (−0.06 [95% CI: −0.09 to −0.04] cm²^.^kg^−^¹^.^m^−^¹, *p* < 0.001, *d* = −1.58) and volume (−0.56 [95% CI: −0.70 to −0.41] cm^2.^kg^−1^, *p* < 0.001, *d* = −2.52). There was no significant difference in peak CSA or volume between uninjured contralateral ST from those with ACLR and healthy control ST.

#### 3.4.2 Middle region

Compared to uninjured contralateral ST, middle region of ACLR ST was significantly smaller in peak CSA (−0.05 [95% CI: −0.07 to −0.02] cm²^.^kg^−^¹^.^m^−^¹, *p* < 0.001, *d* = −1.04) and volume (−0.82 [95% CI: −1.07 to 1.07] cm^3.^kg^−^¹^.^m^−^¹, *p* < 0.001; *d* = −1.60) (Figure 3). Compared to the healthy controls, ACLR ST was also significantly smaller in peak CSA (−0.05 [95% CI: −0.08 to −0.02] cm²^.^kg^−^¹^.^m^−^¹, *p* < 0.001, *d* = −1.16) and volume (−0.73 [95% CI: −1.00 to −0.45] cm^3.^kg^−^¹^.^m^−^¹, *p* < 0.001, *d* = −1.81). There was no significant difference in peak CSA or volume between uninjured contralateral ST from those with ACLR and healthy control ST.

#### 3.4.3 Distal region

Compared to uninjured contralateral ST, distal region of ACLR ST was significantly smaller in peak CSA (−0.04 [95% CI: −0.06 to −0.01] cm²^.^kg^−^¹^.^m^−^¹, *p* = 0.005, *d* = −0.76) and volume (−0.26 [95% CI: −0.41 to −0.12] cm^3.^kg^−^¹^.^m^−^¹, *p* = 0.001, *d* = −0.91) (Figure 3). Compared to the healthy controls, ACLR ST was also significantly smaller in peak CSA (−0.04 [95% CI: −0.06 to −0.01] cm²^.^kg^−^¹^.^m^−^¹, *p* = 0.005, *d* = −1.00) and volume (−0.21 [95% CI: −0.34 to −0.09] cm^3.^kg^−^¹^.^m^−^¹, *p* = 0.002, *d* = −1.15). There were no significant differences in any region in ST peak CSA or volume between health contralateral limb in ACLR and healthy control.

### 3.5 Impact of tendon regeneration on muscle morphology

**Figure 4:**
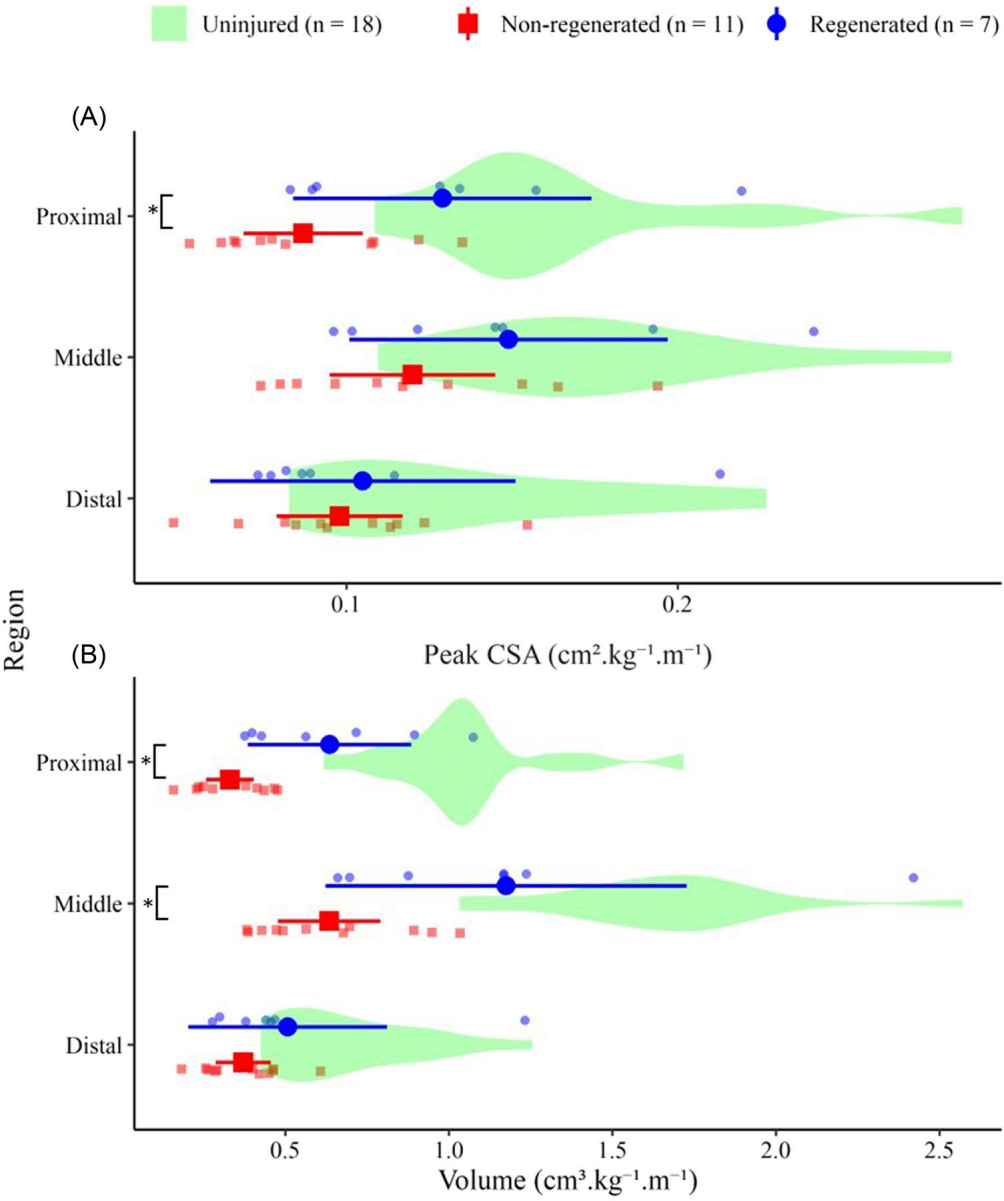
(A) Normalized peak muscle cross‐sectional area (CSA), and (B) normalized muscle volume of proximal (100%–66%), middle (<66%–33%), and distal (<33%–0%) regions of semitendinosus (ST) in anterior cruciate ligament reconstruction (ACLR) participant (nonregenerated, Regenerated). Nonregenerated sub‐group (red) were defined as having no signs of harvested tendon regeneration. Regenerated sub‐group (blue) defined as harvested tendon regeneration. Uninjured contralateral limb of ACLR cohort (green) displayed as a distribution of frequency. Data are presented as mean ± 95% confidence interval. *p < 0.05 compared to regenerated limb subgroup. [Color figure can be viewed at wileyonlinelibrary.com]

Some 39% (7/18) of ACLR ST had tendon regeneration. Compared to ST without regeneration, regenerated ST muscle was significantly longer (10.10 [95% CI: 6.10–14.10] cm^.^m^−^¹; *p* < 0.001; *d* = 2.59) and had greater total muscle volume (0.98 [95% CI: 0.17–1.80] cm^3.^kg^−^¹^.^m^−^¹; *p* = 0.02; *d* = 1.24). Regionally, regenerated ST had significantly greater peak proximal CSA (0.04 [95% CI: 0.005–0.08] cm²^.^kg^−^¹^.^m^−^¹, *p* = 0.03, *d* = 1.16), as well as proximal (0.31 [95% CI: 0.11–0.50] cm^3.^kg^−^¹^.^m^−^¹, *p* = 0.004, *d* = 1.63) and middle (0.54 [95% CI: 0.12–0.96] cm^3.^kg^−^¹^.^m^−^¹, *p* = 0.02; *d* = 1.32) volumes compared to nonregenerated ST (Figure 4).

## 4 DISCUSSION

The primary finding of this study was lower bilateral ST shape similarity in ACLR participants compared to healthy controls, particularly in the distal muscle region. In contrast, deficits in ACLR ST peak CSA and volume were greatest in proximal and middle regions. In ST without distal tendon regeneration, regardless of the method of analysis (i.e., shape similarity or discrete measures), morphological maladaptation was more pronounced. The ST shape following ACLR might alter force transmission and distribution through the muscle, which may have implications for load sharing amongst hamstrings and reduced knee flexor strength‐endurance. Effects of ST shape on lower‐limb function are not yet understood, however, this study provides the first three‐dimensional analysis of ST post‐ACLR.

Consistent with our first hypothesis, bilateral shape similarity was lower in ACLR compared to healthy control ST. Indeed, the greatest shape differences were in the distal ST of the ACLR limb, adjacent to where tendon harvesting occurred. No previous studies have examined ST shape following ACLR, making direct comparison challenging. In principle, ST shape may influence force transfer to bone due to its influence on muscle's line of action. Nonuniform changes in muscle shape, which we have identified to occur in this study, may also contribute to altered coordination and load sharing amongst synergist knee flexors (e.g., hamstrings). Consistent with this notion, Tampere et al.^29^ found male soccer players who returned to sport following ST ACLR had relatively greater biceps femoris and relatively less ST activity during a fatiguing leg curl test compared to uninjured controls. Previous observations in healthy uninjured soccer players suggested reduced reliance on ST associated with reduced knee flexor strength‐endurance^29^ and a significantly greater risk of hamstring strain injury in the subsequent 18 months.^29^, ^30^ Furthermore, patients who receive a semitendinosus autograft ACLR are at ~2.5 times greater risk of ipsilateral ACL reinjury compared to other ACLR autograft choices.^31^, ^32^, ^33^ Prospective studies are needed to determine if bilateral ST shape changes due to ACLR influence rates of subsequent hamstring or ACL injury.

Confirming our second hypothesis, tendon regeneration appears to preserve ST muscle shape in ACLR participants. Compared to those with nonregenerated ST muscles, regenerated ST muscle had significantly less bilateral shape difference. In our ACLR cohort, 39% had regenerated tendon, which is considerably lower than the ~70% previously reported.^4^ We defined tendon regeneration as having occurred if neo‐tendon was visible below the distal muscle‐tendon junction and traced to the level of the femoral epicondyle. This definition is consistent with recent studies assessing hamstring tendon regeneration following ACLR.^17^, ^34^, ^35^ Tendon regeneration following ACLR may be necessary for effective force transmission (neglecting role of lateral force transmission) and modulates the extent of morphological maladaptation following surgery.

Compared to ST in healthy contralateral limb, ACLR ST was significantly shorter and smaller (both peak CSA and volume). Shortening of ST following ACLR has been reported in comparison to preoperative,^17^ uninjured contralateral,^5^, ^36^ and healthy controls.^6^ The shortened ST is likely due to muscle‐belly retraction immediately following harvesting and proximal migration of the distal musculotendinous junction.^37^ Postoperative ST atrophy may shorten its fascicle length thereby reducing active operating range.^38^ Reduction of physiological CSA will lower muscle force capacity and therefore impair ST generated posterior tibial drawer which supports the ACL.^39^ The greatest deficits in ST peak CSA and volume were found in proximal and middle regions, as these regions comprise the majority of ST muscle mass (based on uninjured and healthy control data) and can be expected to experience the greatest postoperative loss in absolute terms. Interventions aimed at promoting hypertrophy in the ST or its agonists might be an important component of postoperative rehabilitation.^40^ As hypothesized, we observed significantly longer muscle with greater volume in those with ST tendon regeneration post‐ACLR compared to those without. Regionally, ACLR limbs with tendon regeneration had significantly greater proximal peak CSA and greater muscle volume in proximal and middle regions compared to those without regeneration. This highlights the importance of the tendon preserving surgical techniques and re‐affirms the need to study the mechanism of tendon regeneration following graft harvesting.

This study had limitations that should be considered. First, the retrospective design means we cannot determine whether observed differences in ST morphology among ACLR, uninjured contralateral, and healthy controls were due to graft harvest or were present at ACL injury. However, healthy controls had no significant between‐limb differences in ST shape or discrete morphology, suggesting observed interlimb differences in ACLR are unlikely to have predated injury. Second, we did not exclude participants with a history of hamstring strain, or other significant lower limb injury that did not require surgery, which may have confounded our results as hamstring atrophy and remodeling has been observed 5–23 months following hamstring strain.^40^, ^41^ Third, we did not account for previous training history of participants, which may contribute to altered ST shape and morphology.^27^, ^42^ However, training effects on ST are likely overshadowed by the substantial morphological changes following ACLR. Fourth, due the retrospective nature of the analysis, a standardized follow‐up time was unable to be implemented, this may have influenced the rate of tendon regeneration observed within this study. Fifth, although great effort and instruction were taken to ensure that positioning was maintained throughout the MRI procedure, interlimb differences in limb positioning was not quantified and could therefore introduce error into the shape analysis of the ST muscle. However, given both groups and legs would have been exposed to the same conditions, it is unlikely to affect bilateral or between‐group comparisons, but might affect absolute values of shape variation. Finally, assessing functional implications of altered ST morphology was beyond the scope of this study. Future studies should aim to understand the consequences of ACLR tendon harvesting on the ST function during dynamic “high risk” movements and if muscle shape change and regional morphology are correlated to lower secondary ACL and hamstring injuries. This is in part due to the ST being an important knee flexor and antagonist of external valgus torque,^10^, ^11^, ^12^ possibly protecting the ACL during complex movement tasks such as landing and cutting maneuvers. Further analyzes are warranted to examine the influence of shape change of ST on the internal muscle architecture parameters such as fascicle lengths and arrangement using imaging modalities such as diffusion tensor imaging or ultrasound.

## 5 CONCLUSION

Shape similarity analysis provided unique perspective on ST morphology post‐ACLR, highlighting shape differences compared to healthy ST in the distal region not captured by traditional discrete morphology measures. Different shape following ACLR might alter force transmission and distribution in the ST, which may have implications for load sharing amongst the hamstrings and knee flexor strength‐endurance. Tendon regeneration in participants with ACLR was shown to result in both improved bilateral shape similarity regional morphology of the ST. Future work might focus on methods to reduce tendon harvest induced muscle morbidity and promote tendon regeneration.

## AUTHOR CONTRIBUTIONS

William du Moulin: Study design, data analysis, and manuscript development. Matthew Bourne: Study design, study supervision, and manuscript development. Laura Diamond: Study design, study supervision, and manuscript development. Jason Konrath: Patient recruitment and manuscript development. Christopher Vertullo: Patient recruitment, manuscript development, study oversight. David Lloyd: Patient recruitment and manuscript development. David Saxby: Recruitment, study design, manuscript development, and study oversight.
